# Supplementary material for: Comparative multiomics analysis of cell physiological state after culture in a basket bioreactor
Source: Sci Rep. 2022 Nov 23;12:20161. doi: 10.1038/s41598-022-24687-4 (PMC9686226; doi:10.1038/s41598-022-24687-4)
Supplement: Supplementary file 1 — Supplementary Information 1. [file 41598_2022_24687_MOESM1_ESM.zip › raw data/Transcriptomic raw data/7.AS/2.dasplot/TE1vsTC1/SE/5_ELF3_NW_023666055.1_28179144_28179314_-@NW_023666055.1_28178256_28178477_-@NW_023666055.1_28177998_28178090_-.pdf]

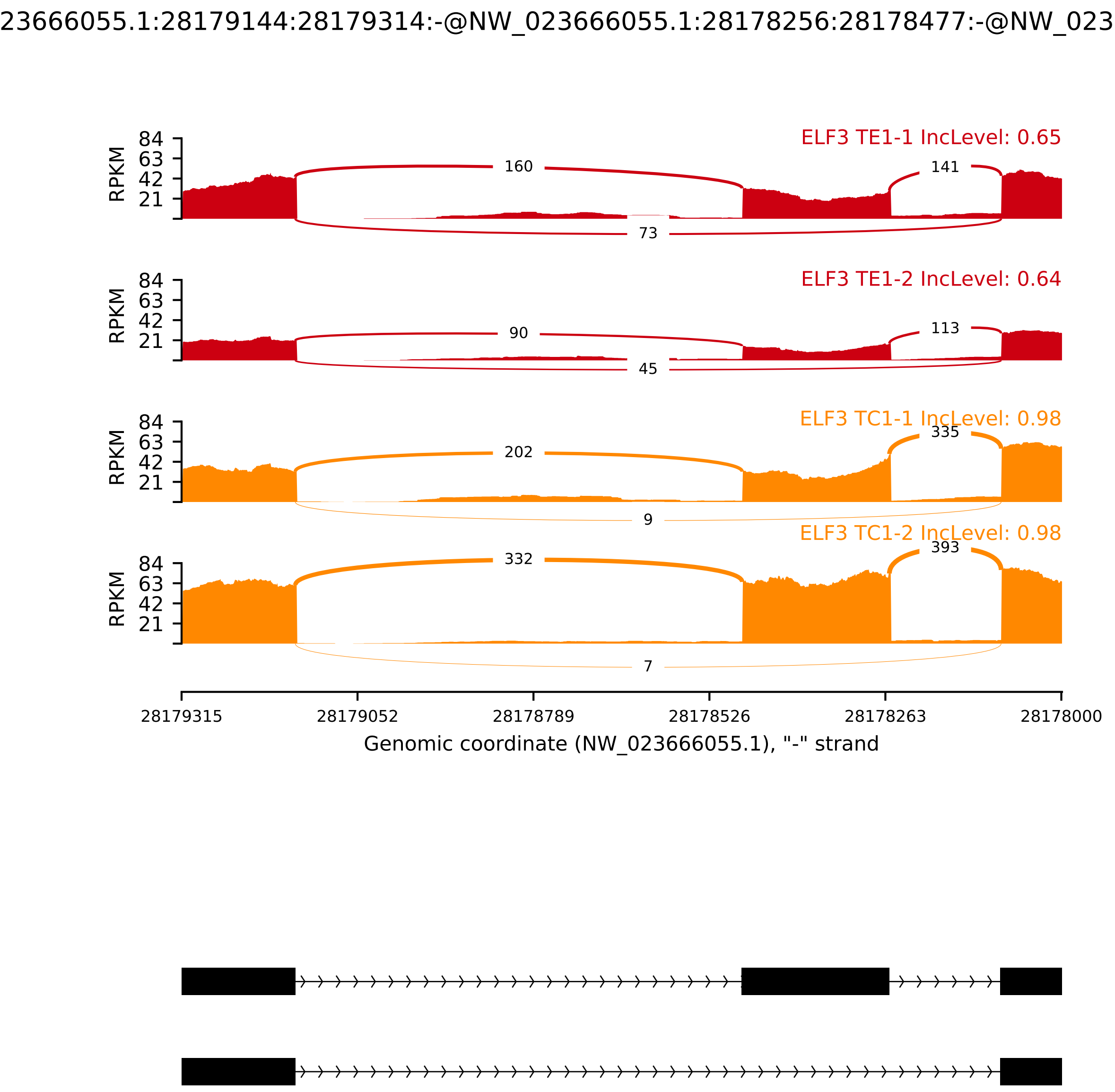
23666055.1:28179144:28179314:-@NW_023666055.1:28178256:28178477:-@NW_023666055.1:28177998:2817
ELF3 TE1-1 IncLevel: 0.65
160
141
73
ELF3 TE1-2 IncLevel: 0.64
90
113
45
ELF3 TC1-1 IncLevel: 0.98
202
335
9
ELF3 TC1-2 IncLevel: 0.98
332
393
7
RPKM
84
63
42
21
28179315
28179052
28178789
28178526
28178263
28178000
Genomic coordinate (NW_023666055.1), "-" strand
